# The development of a DVD to aid patients’ understanding of surgical breast reconstruction clinical trials: QUEST Trials A & B

**DOI:** 10.1186/1745-6215-12-S1-A85

**Published:** 2011-12-13

**Authors:** Judith Mills, Maria Emson, Judith M Bliss, Zoe E Winters

**Affiliations:** 1 Clinical Trials & Statistic Unit (CTSU-ICR), The Institute of Cancer Research, Sutton, Surrey, UK; 2 University of Bristol, Department of Clinical Science South Bristol, Bristol, UK

## Introduction

There is a pressing need for further clinical evidence to better inform both patients and clinicians when recommending the optimal type and timing of breast reconstruction. The QUEST trials are unique; the first surgical trials comparing different types (A) and timing (B) of Latissimus dorsi (LD) reconstruction with a primary outcome of quality of life (QoL). Surgical trials are challenging, therefore necessitating a feasibility study to assess the acceptability of randomisation from both the perspectives of the patients and healthcare professionals. It was decided to develop a DVD to compliment the patient information sheet, in order to help patients understand the concepts of the clinical trials, surgical techniques, randomisation and clinical equipoise.

## Methods

All the women filmed in the DVD had a prior breast reconstruction and were invited to participate in a Q&A session with the Chief Investigator (CI) of the QUEST trials. The CI and 2 breast care nurses were also interviewed about the surgical techniques, complications and side-effects and the time taken to return to normal everyday activities after a breast reconstruction. All women will be given a short questionnaire to complete, assessing their level of understanding of randomisation and clinical equivalence irrespective of their entry into the QUEST trial.

## Results

Filming took place over 1 day in Bristol and editing over a further 2 months. Drawings were produced to pictorially explain clinical equivalence, randomisation and the different types of LD reconstruction. The DVDs and the patient information sheets will be given to all women who are eligible and interested in the QUEST trials. We will be evaluating the impact of this DVD on patient recruitment by both quantitative (questionnaires) and qualitative (interviews) methodology.

## Conclusions

It proved feasible to develop a patient targeted DVD based on the experience of patients and healthcare professionals to enable potential QUEST participants to make fully informed decisions.

